# Pseudoaneurysm of the Abdominal Aorta at the Celiac Trunk After Penetrating Trauma

**DOI:** 10.7759/cureus.17111

**Published:** 2021-08-11

**Authors:** Camellia Nabati, Stephanie Yee, Paul Hanna, Scott Wessner, Robert V Madlinger

**Affiliations:** 1 Surgery/Trauma, St. Joseph's University Medical Center, Paterson, USA; 2 Surgery, St. Joseph's University Medical Center, Paterson, USA; 3 General Surgery, St. Joseph's University Medical Center, Paterson, USA; 4 Trauma/Surgery/Surgical Critical Care, St. Joseph's University Medical Center, Paterson, USA

**Keywords:** dissection, superior mesenteric artery, sma, pseudoaneurysm

## Abstract

We report the case of a 33-year-old man who had received multiple gunshot wounds to the abdomen; consequently, he was diagnosed with a traumatic dissection of the abdominal aorta at the level of the superior mesenteric artery (SMA) extending to just below the renal arteries with a posterior pseudoaneurysm of the aorta. He had wounds in the right upper quadrant and in the left lower back. He demonstrated signs of peritonitis for which he was taken to the operating room for exploratory laparotomy. A right common iliac to SMA bypass with a 7-mm ringed polytetrafluoroethylene (PTFE) graft was created. The celiac trunk was then ligated, and through the right groin sheath, a thoracic endograft stent (Cook Medical, Bloomington, IN) was inserted at the level of the thoracic aorta with resolution of the blood flow to the aorta, visceral and iliac arteries, as well as retrograde flow into the bypass graft. The literature on traumatic abdominal aortic pseudoaneurysm was reviewed, and based on that, we believe this report describes a unique case of a traumatic aortic pseudoaneurysm at the level of the celiac trunk, as well as our operative approach.

## Introduction

While only 1-2% of traumatic injuries are vascular in nature, vascular injuries account for more than 20% of all trauma-related deaths [1]. Although traumatic aortic pseudoaneurysms are a rare occurrence, case reports on its management are available in the literature, and several techniques related to its management have been described. However, this case report is unique in that it does not deal with the management of an aortic pseudoaneurysm from penetrating trauma directly in the vicinity of celiac or mesenteric takeoff. We describe a remarkable case of a young male with a traumatic pseudoaneurysm directly posterior to the celiac and superior mesenteric artery (SMA) takeoffs.

## Case presentation

A 32-year-old male with no significant medical history presented to the St. Joseph’s University Medical Center after sustaining multiple gunshot wounds to the abdomen. On arrival at the trauma bay, the patient complained of abdominal pain. On admission, his vital signs were notable for a heart rate of 90 beats per minute and blood pressure of 169/85 mmHg. On examination, the patient was neurologically intact, and cardiac and pulmonary exams were normal. Abdominal examination was notable for peritonitis and a gunshot wound to the anterior right abdomen, and a second wound approximately 4 cm to the left of the midline at the level of L1. Peripheral pulses were intact. The initial chest radiograph showed no abnormality. The abdominal radiograph showed a bullet overlying the right cardiophrenic angle as well as subtle fractures of L1 and L2. The initial focused assessment with sonography in trauma (FAST) exam was negative. On clinical examination, due to peritonitis along with the suspected trajectory of the gunshot wound, the patient was taken to the operating room for further exploration.

**Figure 1:**
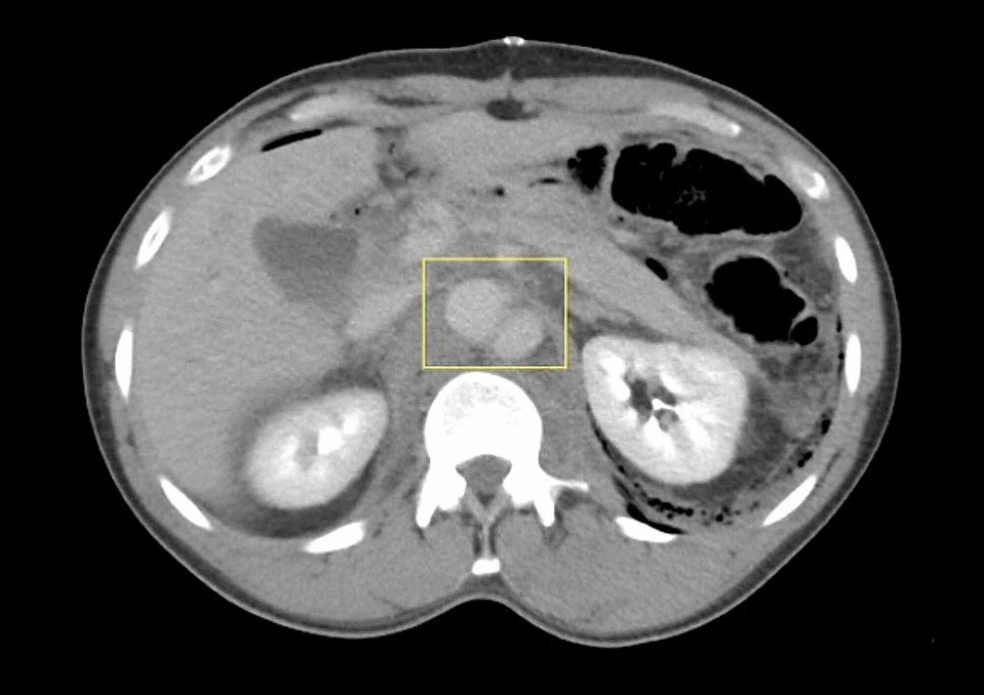
Axial CT showing aortic dissection at the level of the SMA extending to the renal arteries (box) CT: computed tomography; SMA: superior mesenteric artery

**Figure 2:**
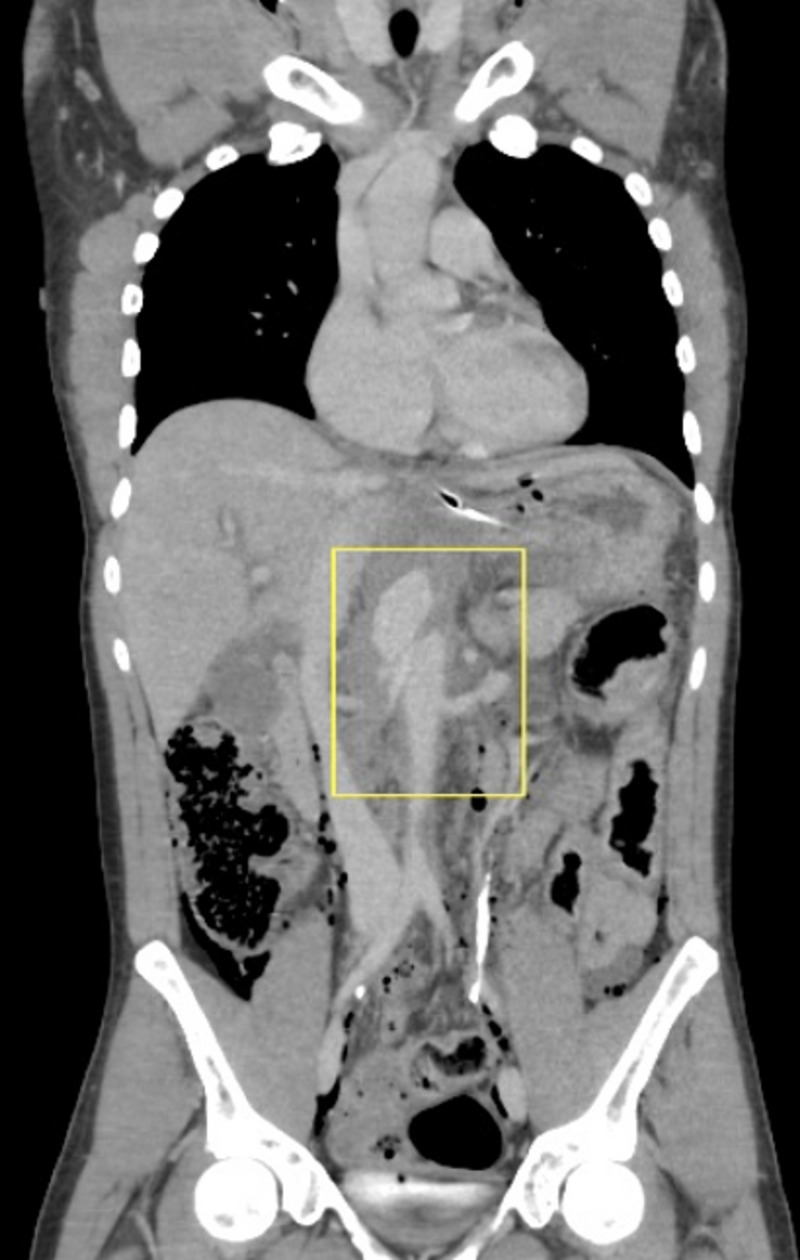
Coronal CT showing aortic dissection at the level of the SMA (box) CT: computed tomography; SMA: superior mesenteric artery

The patient underwent an exploratory laparotomy with right and left medial visceral rotations. There was a wound through the anterior right lobe of the liver just to the right of the falciform ligament for which Floseal® (Baxter International. Deerfield, IL) was applied. Otherwise, there was no evidence of hemorrhage, and no other visceral or vascular injury in the peritoneal or retroperitoneal cavity was noted. An upper endoscopy was also performed, which showed no injury. The patient was stable and there was no apparent presence of any intra-abdominal or immediately visible retroperitoneal injury; hence, the patient was taken for a CT scan of the chest, abdomen, and pelvis. This imaging was notable for dissection of the abdominal aorta at the level of the SMA extending to just below the renal arteries with a posterior pseudoaneurysm of the aorta of ~1.9 x 1.5 x 2.5 cm without active extravasation (Figures 1, 2). Additionally, there was multifocal retroperitoneal hemorrhage as well as a comminuted fracture of the L1 pedicle and transverse process fracture of L2. 

**Figure 3:**
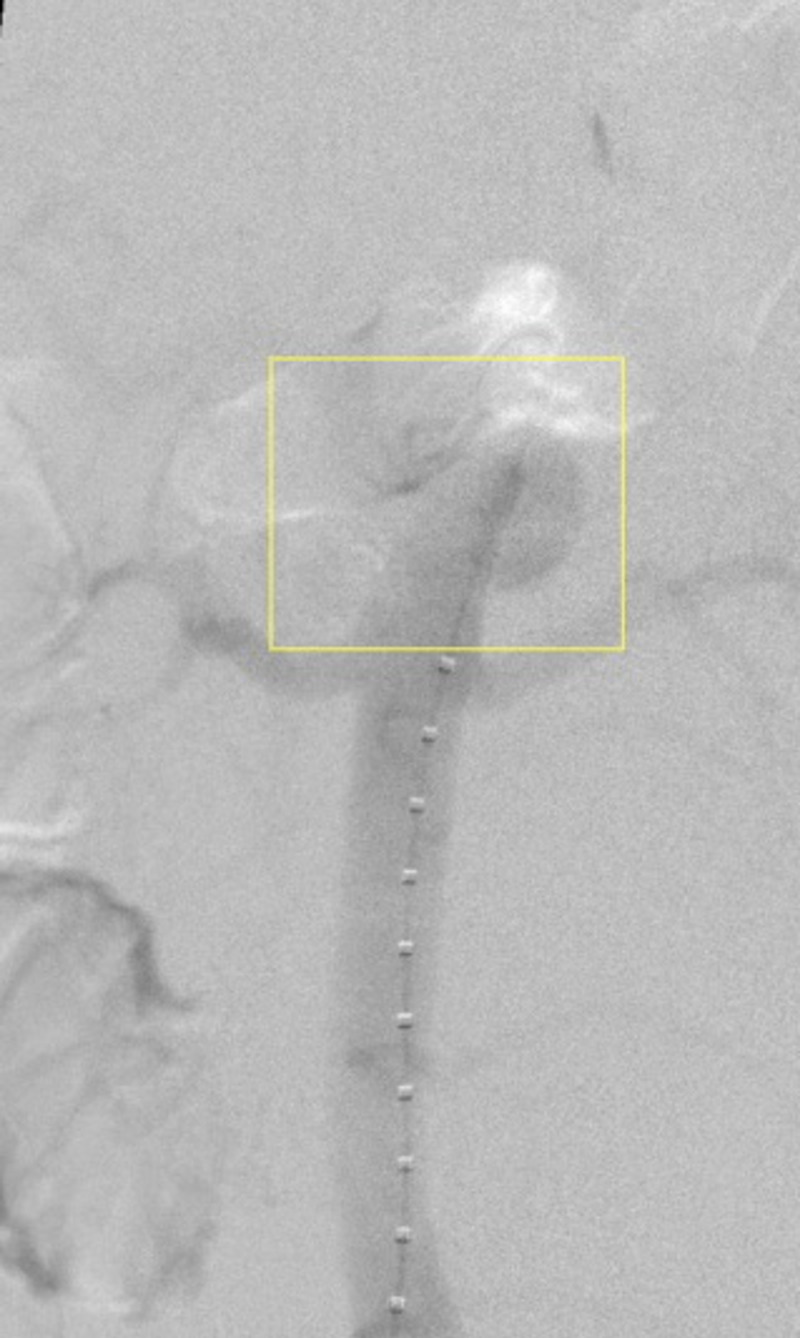
Aortogram showing aortic pseudoaneurysm at the level of the SMA (box) SMA: superior mesenteric artery

**Figure 4:**
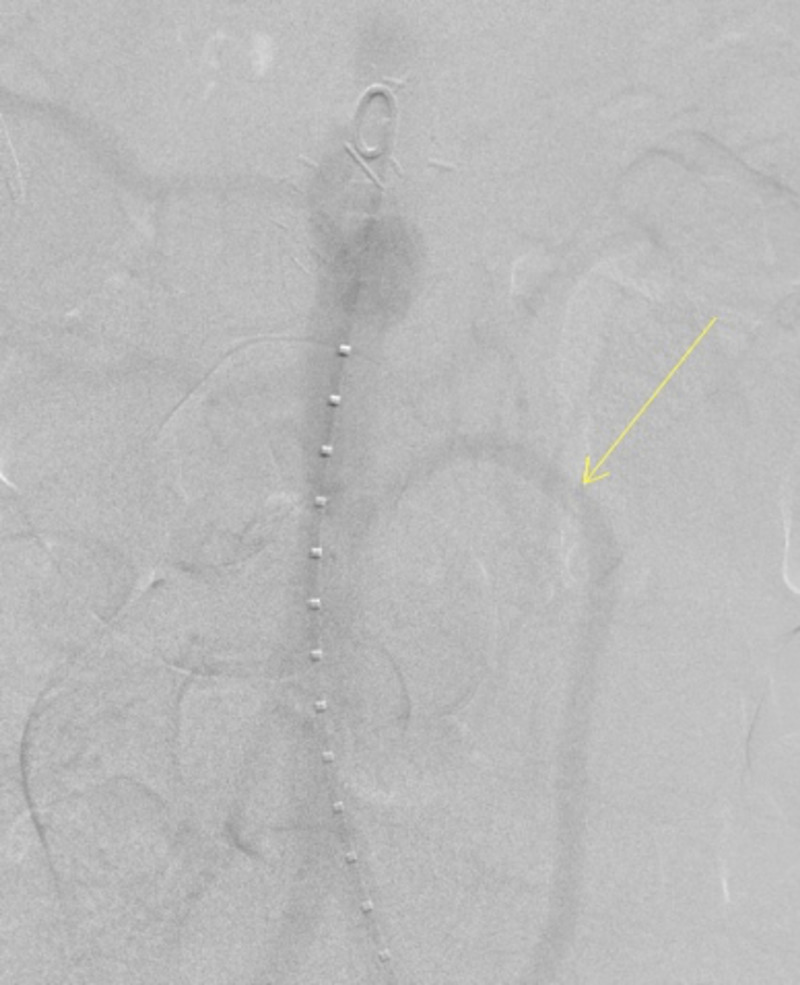
Aortogram showing the patent and retrograde flow through the right common iliac to SMA bypass (arrow) - view 1 SMA: superior mesenteric artery

**Figure 5:**
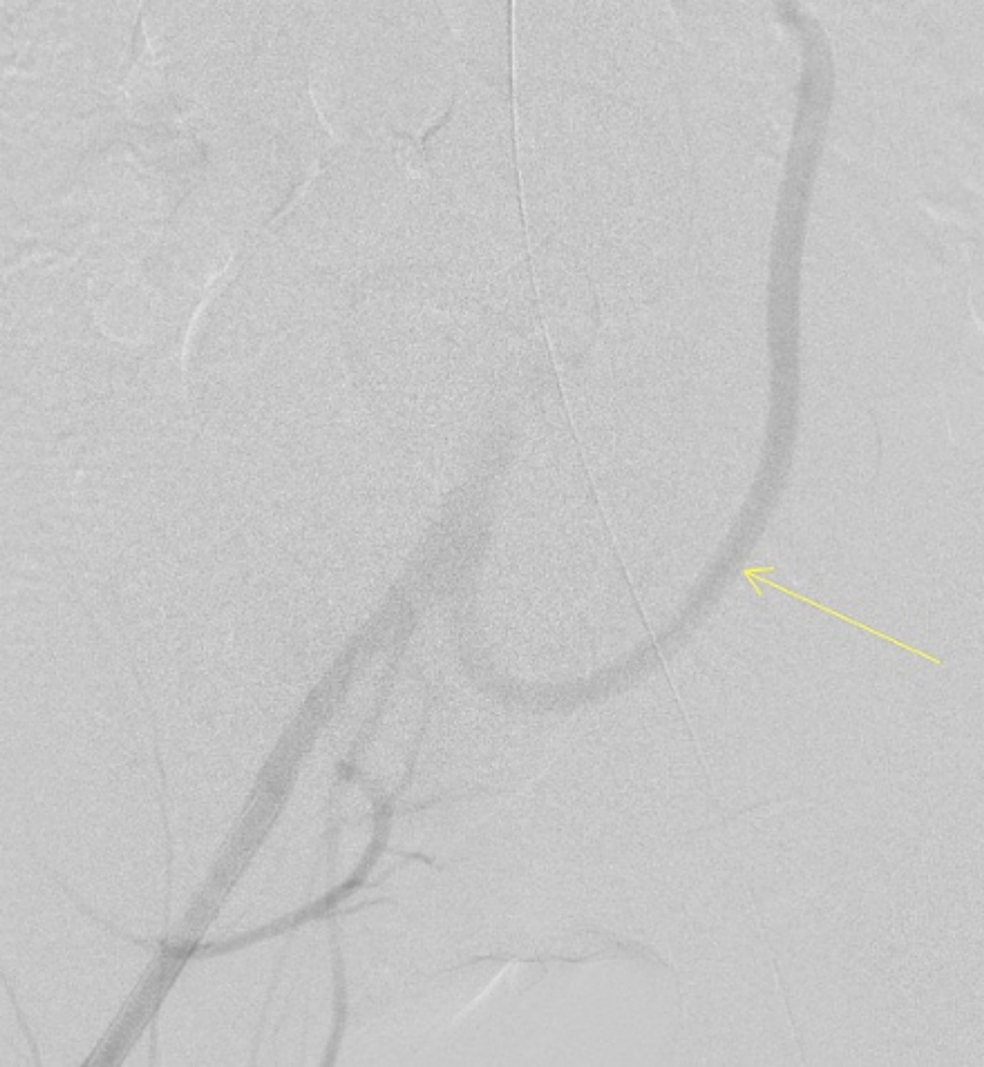
Aortogram showing the patent and retrograde flow through the right common iliac to SMA bypass (arrow) - view 2 SMA: superior mesenteric artery

**Figure 6:**
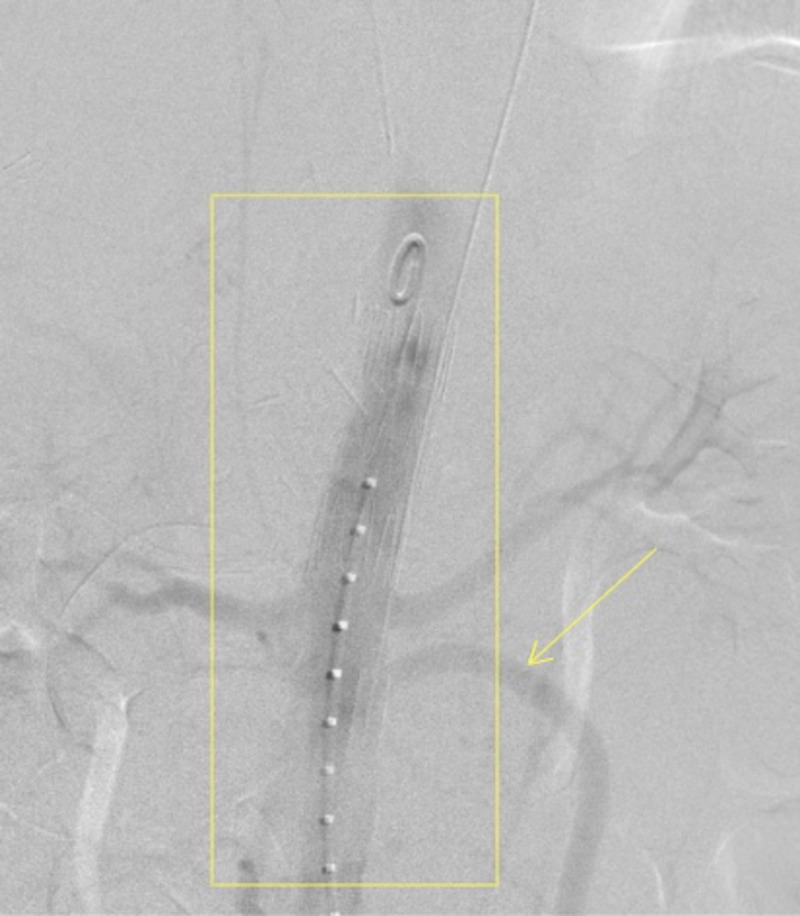
Aortogram showing the aortic endograft stent in place with obliterated pseudoaneurysm (box), along with patent right common iliac to SMA bypass (arrow) SMA: superior mesenteric artery

The patient was brought back to the operating room for evaluation of the aortic pseudoaneurysm. An aortogram was performed, which identified the aortic pseudoaneurysm at the level of the L1 vertebral body, and it was also noted that the SMA was coming off of the celiac trunk (Figure 3). The patient was heparinized and the SMA, as well as the right common iliac artery, were dissected out circumferentially and isolated. A right common iliac to SMA bypass with a 7-mm ringed polytetrafluoroethylene (PTFE) graft was created. The celiac trunk was then ligated after retrograde perfusion of all of its branches was confirmed. A repeat aortogram confirmed that the bypass graft had retrograde filling into the SMA along with celiac branches (Figures 4, 5). Next, through the 22-French right groin sheath, a thoracic endograft stent (Cook Medical, Bloomington, IN) measuring 22 x 58 mm was inserted at the level of the thoracic aorta just above the renal arteries and deployed. A second stent measuring 22 x 39 mm was deployed just superior to it, with a 17-mm overlap. A repeat aortogram was performed, which confirmed that the pseudoaneurysm was obliterated (Figure 6), with continued and adequate blood flow to the aorta, visceral and iliac arteries, as well as retrograde flow into the bypass graft. Closure of the abdomen was not possible due to bowel edema, and an ABThera™ wound vac device (3M, Maplewood, MN) was placed. Postoperatively, the patient had normal and palpable distal pulses throughout.

The patient was evaluated by orthopedics and a decision was made for the nonoperative management of the L1 pedicle and L2 transverse process fractures. The patient had a prolonged hospital course including multiple returns to the operating room for staged abdominal closure, with a partial closure of the abdomen with the placement of VICRYL® mesh and eventual split-thickness skin graft over the abdomen. A Follow-up CT scan of the abdomen and pelvis performed one month postoperatively was notable for a 6 x 8.8 x 16.5-cm collection surrounding the superficial femoral artery (SFA) to the right common iliac artery graft causing a mass effect resulting in right hydronephrosis, along with intestinal ileus. A CT angiogram was also performed to confirm that there was no extravasation from the bypass into the collection. The patient was treated with IV antibiotics for the peri-graft collection and total parenteral nutrition (TPN) with a nasogastric tube (NGT) for intermittent intestinal ileus. A repeat CT scan two months postoperatively redemonstrated the peri-graft collection, which was increased in size to 11 x 7.7 cm, a possible fistula between the small bowel and sigmoid colon, and worsening right hydronephrosis where the distal ureter was no longer visualized. The patient had interventional radiology (IR) drainage of the peri-graft collection; serous fluid was drained and cultures grew *Enterobacter cloacae*. He was started on daptomycin 500 mg intravenously once every 24 hours for 30 days and meropenem 1000 mg intravenously every eight hours for 30 days. In addition, he was provided ciprofloxacin 500 mg one tablet every 12 hours and Bactrim double strength containing 800 mg sulfamethoxazole and 160 mg trimethoprim in tablet form with one tablet every 12 hours, along with Fluconazole 100 mg tablet once a day. All three medications were administered orally. A repeat scan showed persistent peri-graft collection despite the initial drainage. The second drainage was attempted; however, pulsatile blood was aspirated and there was a concern for a leaking aneurysm of the bypass graft, and hence the procedure was aborted.

Repeat CT scan again showed the persistent peri-graft collection, as well as an enterocutaneous fistula (ECF) from the small bowel in the center of the skin graft. On hospital day 94, the patient underwent operative exploration in an attempt to drain the peri-graft collection. The patient had retroperitoneal exploration; once the surface of the suspected peri-graft collection was exposed, a Doppler ultrasound was used and the collection was noted to have increased vascularity with dense fibrotic adhesions, it was deemed unsafe for further exploration and the procedure was aborted. An Eakin pouch (6.9 x 4.3 inches; TG Eakin, Comber, UK) was placed over the fistula, which covered the skin graft and extended onto his native skin. There was a small amount of slightly thick, mustard-colored drainage present in the bag. When fistula output became minimal, the Eakin pouch was replaced with Convatec one-piece cut-to-fit ostomy pouch (ConvaTec Group, Deeside, UK). His WBC trended down, and he was placed on a regular diet with boost supplement and his fluid balance and electrolyte were carefully monitored. The patient was eventually started on an enteral diet and continued on TPN for malnutrition; he required an IR-guided nephrostomy tube for the right hydronephrosis and was continued on antibiotics as mentioned above for the peri-graft collection. The ECF remained to have low output and was treated with local wound care. A repeat CT scan showed an interval decrease in the peri-graft collection. In summary, the patient had a prolonged hospital course complicated by a peri-graft collection, and intestinal ileus along with an enterocutaneous fistula, but ultimately became stable and his fistula resolved upon discharge. On hospital day 117, under the custody of law enforcement, he was discharged and instructed to take the three previously mentioned oral antibiotics at home.

## Discussion

Pseudoaneurysms of the abdominal aorta are extremely rare and account for only 1% of all abdominal aneurysms [2]. It is often secondary to aortic surgery or penetrating trauma and is characterized by complete disruption of the aortic intima and media, leaving the vascular integrity to be maintained by the adventitia and associated with hematoma formation. The first published report of traumatic abdominal aortic pseudoaneurysm was in 1920 [3]. Since then, a literature review of 22 cases has reported that 79% of cases resulted from penetrating trauma, with 60% located above the renal arteries [4]. While aortic rupture can initially cause death by exsanguination, the development of a pseudoaneurysm may allow long-term survival, as it contains the blood loss within the adventitia or surrounding retroperitoneal tissues [5]. Pseudoaneurysms may initially be life-saving, but spontaneous rupture and death can occur at any time.

The clinical presentation of abdominal aortic pseudoaneurysm varies significantly, and because of this, not all cases are diagnosed at the time of onset. Pseudoaneurysms can be asymptomatic or can present with pain, palpable masses, bruits, upper gastrointestinal bleeding, obstructive jaundice, acute thrombosis of bilateral common iliac arteries, or many other occlusive sequelae [4-8]. In many cases, the diagnosis of an aortic pseudoaneurysm is difficult to establish and may cause a delay in treatment. In general, imaging modalities such as abdominal ultrasound, CT, or angiography are used for diagnosis.

Expeditious repair of this injury is necessary as the risk of spontaneous rupture is associated with high mortality. Given the rarity of traumatic aortic pseudoaneurysms, there is scarce data in the current literature on the optimal management of this type of injury. More specifically, there is no report of the management of a traumatic pseudoaneurysm at the level of the celiac trunk, which occurred in our case. The case reports published so far have been limited to the management of traumatic aortic pseudoaneurysms. The surgical options performed vary from graft replacement, aortorrhaphy, and aortoplasty with the patch, to extra-anatomic bypass [4]. Different case reports have proposed various approaches due to varying factors related to the site and size of pseudoaneurysms, the number of defects, condition of the abdomen, and presence of infection. For example, Bechara-Zamudio et al. have described a case of a patient who sustained a traumatic inframesenteric aortic pseudoaneurysm that was successfully treated with balloon-expandable bifurcated endoprosthesis [9]. In their case, the patient had undergone surgery nine months previously due to multiple gunshot wounds with bowel resections, and because of a “hostile abdomen,” a decision was made to perform an endovascular intervention. Our patient had sustained a gunshot wound, which had resulted in a pseudoaneurysm at the level of the SMA. Interestingly, our patient was found to have a celiacomesenteric trunk, where the SMA and celiac artery have a common trunk. This is a rare vascular anatomical variant that appears in only 1.5% of the population [10]. Our operative decision was to perform an SMA to the right common iliac bypass PTFE graft with an aortic stent to obliterate the pseudoaneurysm. This decision was based on the vicinity of the aortic pseudoaneurysm to the takeoff of the celiac trunk. Current literature provides no guidance or data on the management or outcomes of pseudoaneurysms at the level of the celiac or mesenteric takeoff.

## Conclusions

Aortic pseudoaneurysms carry a risk of spontaneous rupture associated with high mortality; due to the rarity of this injury, there is significant variability in its management. We described a unique case of a traumatic aortic pseudoaneurysm at the level of the celiac trunk as well as our operative approach. Further studies are required to gain more insight into the optimal management of traumatic abdominal aortic pseudoaneurysm.
